# Emergency cardiac surgery and heparin resistance in a patient with essential thrombocythemia

**DOI:** 10.1186/s40981-016-0063-4

**Published:** 2016-11-09

**Authors:** Mika Nakanishi, Eri Oota, Takehiro Soeda, Kaoru Masumo, Yukihiko Tomita, Takeshi Kato, Toshihiro Imanishi

**Affiliations:** Department of Anesthesia, Osakafu Saiseikai Noe Hospital, Fruichi 1-3-25, Joto-ku, Osaka City, Osaka Japan

**Keywords:** Essential thrombocythemia, Heparin resistance, Emergency cardiac surgery

## Abstract

A 66-year-old man with thrombocytosis was brought to our hospital to undergo removal of a left ventricular thrombus. He had developed cerebral infarction 6 days before presenting to the hospital and suffered from right incomplete hemiparalysis. Blood tests on admission revealed his platelet count to be 124.3 × 10^4^/μl. The urgent removal operation was performed under general anesthesia. For carrying out extracorporeal circulation (ECC), approximately three times as much heparin as expected was needed, as well as antithrombin III (AT III) administration. This met the definition of heparin resistance. The thrombus was removed and surgical left ventricular reconstruction was performed. Aspirin and warfarin were initiated on postoperative day 5. A bone marrow biopsy was performed on postoperative day 8, which revealed hypercellular marrow with megakaryocyte proliferation, and the patient was diagnosed as having essential thrombocythemia (ET). Although hydroxycarbamide administration started on postoperative day 10, his platelet count increased to 290.7 × 10^4^/μl on postoperative day 13. The counts descended gradually, and on postoperative day 34, his platelet count reached the normal range and he was discharged from the hospital. In the perioperative period, his new neurologic abnormality did not appear. Addition of heparin, administration of AT III, and coating the cardiopulmonary bypass circuit with heparin or macromolecular polymer prevented clot formation and enabled safe ECC in a patient with ET and a high platelet count.

## Background

Essential thrombocythemia (ET) is classified as a myeloproliferative neoplasm. The characteristics are megakaryocyte proliferation in the bone marrow, continuously very high platelet counts (>45 × 10^4^/μl), and morphological abnormalities of platelets. ET is ruled out if there are morphological abnormalities of the red or white blood cells. Progression of ET causes both thrombotic and hemorrhagic diathesis. Thrombotic events occur more often than hemorrhagic events, and thrombi form more frequently in arteries than in veins. Effective treatments are anticoagulant therapy and some drugs that reduce platelet numbers, such as hydroxyurea, anagrelide, Janus kinase 2 (JAK2) inhibitors, and α-interferon [1].

There are no clear guidelines on the pre- or postoperative management of patients with ET undergoing cardiac surgery. There have been some case reports on patients with ET who underwent cardiac surgery under cardiopulmonary bypass (CPB). In two reports, the platelet count was reduced to a normal range using hydroxyurea or α-interferon before surgery was performed [2, 3]. In the other reports, the platelet count was reduced by therapeutic plateletpheresis procedures preoperatively [4, 5]. One case report on urgent cardiac surgery under CPB for a patient with ET and a high platelet count was published in Japanese [6]. We present a case report of a patient with ET and a high platelet count who underwent urgent left ventricular thrombus removal under CPB.

## Case presentation

A 66-year-old man weighing 49.9 kg developed cerebral infarction and right hemiparalysis. Transthoracic echocardiography (TTE) showed globally decreased left ventricular function (visual ejection fraction 30 %) and a left ventricular foreign body. Infectious endocarditis was suspected because he also had a fever and elevated C-reactive protein levels and white blood cell count. Antibiotics were administered, but after 2 days the foreign body grew. His platelet count was 130.6 × 10^4^/μl, which suggested thrombocytosis and left ventricular thrombus. Administration of heparin did not improve the thrombus and 6 days after the onset of cerebral infarction the patient was urgently brought to our hospital to have the thrombus removed.

He explained that he had undergone a procedure for a gastric ulcer 1 year before, but he was not aware of his cardiac abnormality on admission. He suffered from right hemiparalysis. His platelet count was 124.3 × 10^4^/μl, his white blood cell count was 156 × 10^2^/μl, and his red blood cell count was 536 × 10^4^/μl. Abnormal white blood cells were not observed. TTE showed severe hypokinesis and akinesis from the middle to the apical areas of the left ventricle. The visual ejection fraction was approximately 35 %. The left ventricular thrombus was located in the septal segments and was 15.5 × 30.0 mm as measured by TTE.

**Fig. 1:**
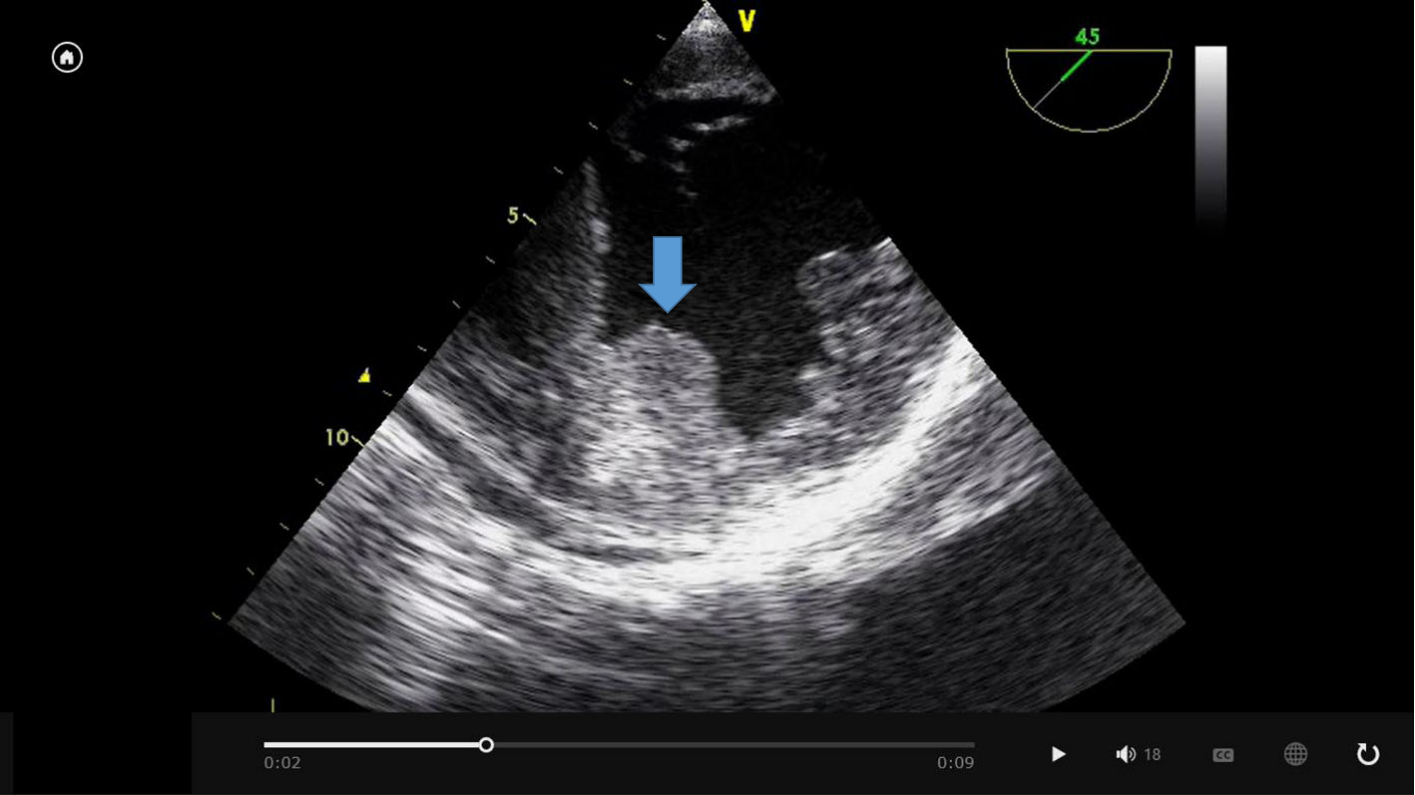
Left ventricular thrombus observed by transesophageal echocardiography under general anesthesia. The thrombus () was mobile and was located in the septal areas

**Fig. 2:**
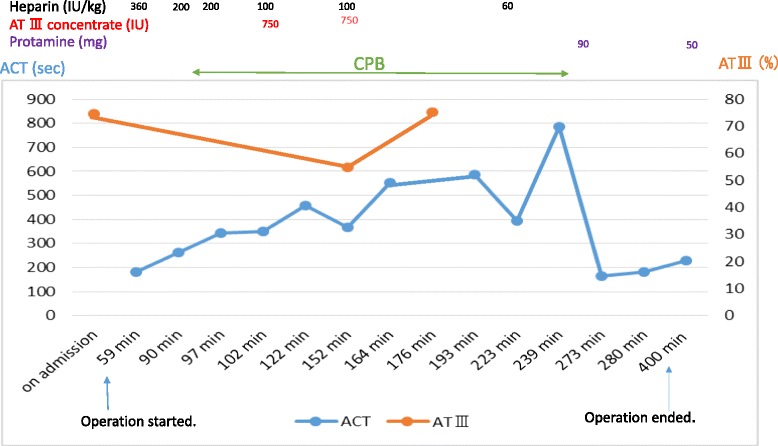
Changes in ACT and AT III. More heparin than expected was needed to carry out extracorporeal circulation. The patient’s AT III levels decreased during surgery, and administration of AT III was needed. Less protamine than expected was needed to neutralize the heparin effect. *ACT* activated coagulation time, *AT III* antithrombin III, *CPB* cardiopulmonary bypass

The urgent removal operation was performed under general anesthesia as soon as hospitalization and several examinations were accomplished. General anesthesia was induced with midazolam, fentanyl, and rocuronium, and maintained with propofol, remifentanil, fentanyl, and rocuronium. Transesophageal echocardiography (TEE) showed a fragile thrombus, not in the left atrial appendage but in the left ventricle (Fig. 1). The activated coagulation time (ACT) was 182 s, and the antithrombin III (AT III) level was 74.6 %. After heparin sodium 360 IU per kg bodyweight (IU/kg) was administered intravenously, the ACT reached 261 s. Heparin sodium 400 IU/kg was added and CPB was started. Despite administration of heparin, the ACT only reached 350 s, which was not long enough to carry out extracorporeal circulation (ECC). AT III concentrate (Neuart^TM^) 750 IU and heparin sodium 100 IU/kg were administered and the ACT extended to 459 s. The patient’s AT III level was examined after administration of the AT III concentrate and was found to be 54.9 %. Further, AT III was administered (750 IU), and the AT III level reached 75.3 % (Fig. 2).

The areas of the left ventricle supplied by the left anterior descending (LAD) artery were thin and a left ventricular aneurysm that contained the thrombus was observed. The thrombus was very fragile and partially liquid. The border between normal tissue and scar tissue of the left ventricle was clear, and the size of the scar was 60 × 40 mm. The thrombus was removed and surgical left ventricular reconstruction was performed. A CPB circuit coated by heparin or macromolecular polymer was used and no clots were seen in the circuit. An intra-aortic balloon pump (IABP) was inserted and used to keep systolic blood pressure low and prevent left ventricular rupture. Protamine sulfate 90 mg (1.8 mg/kg) was administered intravenously to neutralize the heparin, and the ACT returned to 163 s. The regional cerebral oxygen saturation was monitored by near infrared spectroscopy during anesthesia; no obvious changes were observed. The anesthetic duration was 6 h 29 min, the ECC time was 2 h 44 min, and the cross-clamp time was 1 h 59 min. Blood loss was 1401 g. Besides crystalloid solution, 1200 ml of fresh frozen plasma (FFP), 1400 ml of packed red blood cells, and 350 ml of albumin were administered.

The patient entered an intensive care unit after surgery and protamine sulfate 50 mg was administered intravenously because his ACT was still 230 s. The post-surgical platelet count was 55.9 × 10^4^/μl. Bacterial cells were not found in the removed thrombus. The IABP was removed on postoperative day 3, and the patient was taken off mechanical ventilation support on postoperative day 6. Aspirin and warfarin were initiated on postoperative day 5. A bone marrow biopsy was performed 8 days after the surgery, which revealed hypercellular marrow with megakaryocyte proliferation. The patient was diagnosed as having ET. Although hydroxycarbamide administration started on postoperative day 10, the patient’s platelet count increased to 290.7 × 10^4^/μl on postoperative day 13. The count descended gradually, and on postoperative day 27 it reached 100 × 10^4^/μl. In the perioperative period, his new neurologic abnormality did not appear. On postoperative day 28, cardiac angiography revealed that segments 7 and 8 of the LAD artery had 90 % stenosis. Left ventriculography showed akinesis of segments 3 and 6 but no thrombus. On postoperative day 34, the patient’s platelet count was 37.4 × 10^4^/μl and he was discharged from the hospital.

### Discussion

Patients with ET frequently develop thrombotic attacks especially in the brain, heart, and distal arteries [1]. Removal from the heart has risks, and one patient with right atrial thrombus was reported to choose anticoagulant therapy without surgical removal [7]. Our patient developed cerebral infarction, and the size of his thrombus did not reduce despite heparinization; therefore, he decided to undergo a removal operation under CPB. His high platelet count was strongly suspected to be caused by ET before the surgery. However, there was not sufficient time to make a diagnosis or to reduce the platelet count using cytoreductive drugs because the left ventricular thrombus was mobile and the cerebral infarction may have worsened.

A 22-year-old patient with ET was reported to undergo aortic valve replacement under CPB after his platelet count was reduced to 30.1 × 10^4^/μl by treatment with α-interferon [2]. A 64-year-old patient with ET was also reported to receive mitroaortic valve replacement under CPB after his platelet count was reduced to 30 × 10^4^/μl by treatment with hydroxyurea [3]. A 54-year-old patient with ET was reported to have cardiac artery bypass surgery under CPB with a high platelet count: 99 × 10^4^/μl [6]. This case was urgent and there was not sufficient time to reduce the platelet count. In this case, heparin 300 IU/kg was not enough to increase the ACT to longer than 400 s, and eventually 500 IU/kg heparin was needed. The ECC time was 1 h 10 min, and heparin 200 IU/kg was added to keep the ACT longer than 400 s under CPB. However, 1.5 mg/kg protamine was sufficient to neutralize the heparin effect. Perioperative thrombotic events did not occur in this case.

Some abstracts reporting on CPB for patients with ET were presented at academic conferences in Japan. An 81-year-old patient with ET was reported to undergo aortic valve replacement under CPB with a high platelet count of 139.8 × 10^4^/μl [8]. Heparin 300 IU/kg was administered before ECC and nafamostat mesilate 30 mg/h was continuously delivered during the procedure. The CPB circuit was not coated with heparin or other anticoagulant substances. Despite delivering anticoagulant drugs, clots were observed in the reservoir and the aortic filter, which eventually needed to be changed. Another summary reported that a 57-year-old patient with ET had mitral valve plasty under CPB with a platelet count of 121.8 × 10^4^/μl [9]. The dose of heparin administered was not referred to, but delivering heparin continuously into the CPB circuit and using a CPB circuit coated with heparin enabled safe ECC and the reservoir did not need to be changed. In another abstract, the authors reported that a 64-year-old patient who had suspected ET underwent mitral valve plasty under CPB with a platelet count of 73.2 × 10^4^/μl [10]. Heparin (200 IU/kg) was administered before ECC and at that point, the ACT was 236 s. The CPB circuit was coated with anticoagulants, but these were not referred to in detail. During the 2 h 41 min ECC, 50,000 IU heparin was delivered. ACT was checked seven times during CPB, but only twice was it longer than 400 s. The reservoir did not need to be changed, although clots were observed. These case reports demonstrate heparin resistance and the hypercoagulable state and difficulties experienced with CPB in patients with ET.

A 73-year-old man with ET was reported to undergo cardiac surgery after his platelet count had been reduced to a normal range using three therapeutic plateletpheresis procedures [4]. Therapeutic plateletpheresis is a useful method for rapidly reducing the platelet count, but there are no clear guidelines for this procedure for ET patients. The presence of nonfunctional platelets in ET patients poses a problem in establishing a safe preoperative baseline platelet count. Furthermore, it is controversial whether this reduction is adequate for long-term maintenance because the platelet count often rebounds in ET patients [4]. In our case, plateletpheresis might have been effective, but our hospital did not have the necessary equipment.

Thrombocytosis (platelet count >40 × 10^4^/μl) is known to cause heparin resistance [11], as seen in several case reports described above. Heparin resistance is usually defined as the failure to achieve an ACT of 480 s after 450 IU/kg of heparin [12]. Heparin resistance can be caused by mechanisms independent of AT or mechanisms related to AT deficiency. The former mechanisms are nonspecific and poorly defined. The risk factors are abnormal platelets; intravenous administration of nitroglycerin; and increased heparin-binding proteins, such as chemokines, extracellular matrix proteins, growth factors, enzymes, and miscellaneous proteins (factor VIII, von Willebrand factor, and so on). After intravenous administration, heparin can bind to several molecules and platelets that can result in reduced biologic availability. By this mechanism, thrombocytosis might cause heparin resistance. However, the extent to which each of these risk factors contributes to the phenomenon of heparin resistance in the cardiac surgical population is not clear [13]. Clinically significant heparin resistance is suspected to be caused by either AT III deficiency or type 2 heparin-induced thrombocytopenia [14]. Although in many cases heparin addition resolves heparin resistance, sometimes administration of FFP or AT III is necessary for AT III deficiency [12].

AT III is one of the main natural coagulation system inhibitors. It inhibits not only thrombin and factor Xa but also factors IXa, XIa, and XIIa, kallikrein, and plasmin. Antithrombin’s activity is potentiated by heparin and may be a key component of the heparin response. AT III levels are known to decrease after recent or active thrombosis, surgical procedures, disseminated intravascular coagulation, or full-dose heparin administration. Full-dose heparin can lower AT III levels by 30 % within several days, but the levels recover after discontinuing heparin [15].

In our case, AT III administration and approximately three times as much heparin as expected were needed in order to carry out ECC. Possible reasons for the decrease in AT III under CPB were because it was consumed by administration of too much heparin, the surgical procedure itself, and the patient’s hypercoagulable state. Addition of heparin, administration of AT III, and coating the CPB circuit with heparin or macromolecular polymer prevented clot formation and enabled safe ECC. This suggested that in this case, heparin resistance was caused by mechanisms independent of AT and mechanisms related to AT deficiency.

To neutralize the heparin effect, protamine was titrated stepwise while measuring ACT, so the dose of protamine administered in our case was low. We did not want ACT to be completely normalized because of the risk of thrombophilia.

The patient’s platelet count gradually increased after the surgery due to rebound phenomena, but anticoagulant therapy with aspirin and warfarin prevented thrombotic events, and hydroxycarbamide reduced the platelet count to a normal range. Anagrelide would have been inappropriate because of its cardiac depression side effect, and JAK2 inhibitors could not be prescribed under health insurance in Japan. Alpha-interferon was not chosen because it is not a standard treatment for ET.

## Conclusions

Addition of heparin, administration of AT III, and coating the CPB circuit with heparin or macromolecular polymer prevented clot formation and enabled safe ECC in a patient with ET and a high platelet count.
